# Subchronic toxicity evaluation of leaves from rabbiteye blueberry (*Vaccinium virgatum* Aiton) in rats

**DOI:** 10.1016/j.toxrep.2019.03.005

**Published:** 2019-03-18

**Authors:** Wataru Tanaka, Daigo Yokoyama, Yasushi Matsuura, Masahiko Nozaki, Naoya Hirozawa, Hisato Kunitake, Masanobu Sakono, Hiroyuki Sakakibara

**Affiliations:** a Graduate School of Agriculture, University of Miyazaki, 1-1 Gakuen-kibanadai Nishi, Miyazaki 889-2192, Japan; b Miyazaki Prefectural Food Research and Development Center, 16500-2 Sadowara Cho, Miyazaki 880-0303, Japan; c SUNAO Pharma Inc., 2-74 Wachigawara, Miyazaki 880-0023, Japan

**Keywords:** BBL, blueberry leaf, G-CSF, granulocyte colony-stimulating factor, GM-CSF, granulocyte-macrophage colony-stimulating factor, IFN-γ, interferon gamma, IL, interleukin, MIP, macrophage inflammatory protein, NOAEL, no-observed-adverse-effect level, TNF-α, tumor necrosis factor alpha, Blueberry leaf, No-observed-adverse-effect level (NOAEL), Oral toxicity, Subchronic toxicity, Rat

## Abstract

Blueberry leaf may contain multiple compounds with beneficial effects. We conducted a 90-day toxicity study in rats to evaluate the safety of consuming the leaves of rabbiteye blueberry (*Vaccinium virgatum* Aiton; RB species). Powdered leaves were administered daily by oral gavage at doses of 500, 1000, and 2500 mg/kg body weight to male and female Sprague-Dawley rats for 90 days. Treatment did not result in death or changes in the behavior and external appearance of the animals. No alterations were observed in hematological and serum chemical parameters, urinalysis, food consumption, body weight gain, or absolute and relative organ weights at the end of the treatment period, with the exception of some leukocyte percentages in male rats treated with 500 and 1000 mg/kg blueberry leaf powder. The findings indicate that rabbiteye blueberry leaf is safe for consumption and should be investigated as a candidate functional food.

## Highlights

• Blueberry leaf may contain multiple compounds with beneficial effects, but limited about the safety.
• Powdered blueberry leaf has no toxic event at oral dose of daily 500, 1000 and 2500 mg/kg for 90 days in SD rats.
• No significant changes in food consumption, body weight gain and organ weights.
• A daily dose up to 2,500 mg/kg body weight in both the sexes rats may indicate a NOAEL.
• An acceptable daily intake of blueberry leaf powder for humans is calculated to be 25 mg in dry weight per kg body weight.

## Graphical abstract

**Figure:**
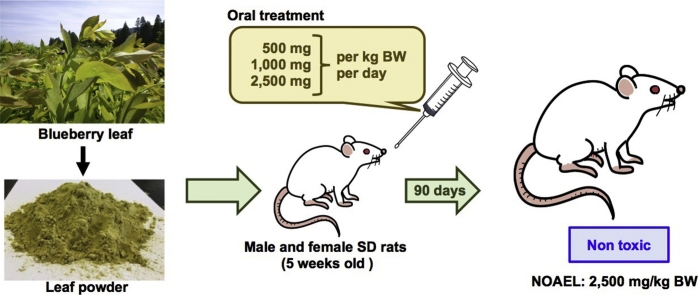

## 1 Introduction

**Fig. 1:**
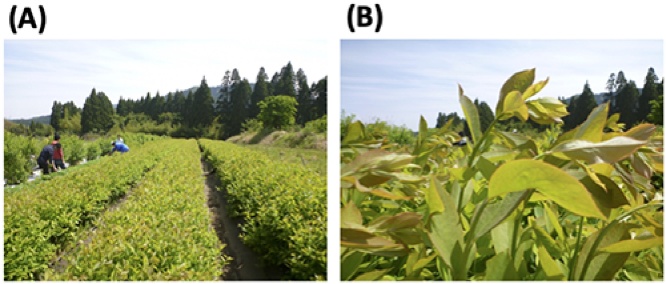
Typical blueberry field. (A) General view; (B) close-up view of the leaves. Photographs by H.S.

Functional foods, also termed “superfoods”, offer health promotion and disease prevention benefits. The consumption of berry fruits may prevent carcinogenesis, liver damage, obesity, and inflammation, improve eyesight, and prevent glaucoma [[1], [2], [3], [4]]. The active compounds in berries are flavonoids, mainly anthocyanins and phenolic acids/esters such as chlorogenic acid [3,5,6]. The leaves of berry-bearing plants, however, are typically ignored and discarded. Berry leaves may contain high quantities of bioactive ingredients such as phenolic acids/esters, flavonoids, and procyanidins [7] that exert anti-diabetic and anti-glycation effects [8,9]. Our group has focused on rabbiteye blueberry (*Vaccinium virgatum* Aiton; RB species; Fig. 1). Previous investigations using extracts from the leaves of rabbiteye blueberry reported anti-hepatitis C virus, anti-hypertensive, hypolipidemic, liver lipid-lowering, adult T-cell leukemia-prevention, and insulin resistance-prevention activities [[10], [11], [12], [13], [14], [15]]. These findings indicate that blueberry leaves may be a candidate functional food.

In the tea industry, tea leaves are discarded as waste following hot-water extraction, although they still contain fat-soluble vitamins, proteins, water-insoluble fibers, and bioactive pigments, which are not easily extracted in hot water [16]. Consuming whole tea leaves rather than their hot-water extracts may therefore be more beneficial [17,18]. To that end, the safety of consuming whole leaves must be established. Although safety data on berry leaf extracts from raspberry plants have been reported [19,20], information is limited about the safety of daily consumption of whole berry leaves. We therefore carried out a 90-day toxicity study of rabbiteye blueberry leaf (BBL) powder in rats.

## 2 Materials and methods

### 2.1 Chemicals

Carboxymethyl cellulose, isoflurane, and Triton X-100 were obtained from Wako Pure Chemical Industries Ltd. (Osaka, Japan). All other reagents were of the highest grade available.

### 2.2 Plant material

Rabbiteye blueberry cultivar Kunisato No. 35 (*Vaccinium virgatum* Aiton) was cultivated in Miyazaki, Japan and taxonomically identified on the basis of morphological characteristics by Prof. Hisato Kunitake (University of Miyazaki, Miyazaki, Japan). The fresh leaves were collected in July 2016 and immediately transported to a tea factory (Slow Life Too Inc., Miyazaki, Japan). BBL powder was produced from the leaves according to the traditional protocol for making Japanese green tea powder. Briefly, the sprigs were removed and the leaves were roasted in a heating cylinder adjusted at 300 °C for 150-sec. The roasted leaves were macerated under room temperature for 20-min and then under hot-air (60 °C) for 20-min, and dried in the rotary pot adjusted at 100 °C. The yield was 33.3%. Following, the leaves were ground to a fine powder. The BBL powders were vacuum-sealed to prevent degradation by air and stored at room temperature.

### 2.3 Institutional approval of the study protocols

All procedures were conducted according to the guidelines for the care and use of laboratory animals of the University of Miyazaki (Miyazaki, Japan). The study protocol was followed the modified methods reported by some research groups [[21], [22], [23]]. In these reports, safety assessments of foods and dietary ingredients were evaluated. Additionally, they were modified methods according to the OECD 408 guideline “Repeated Dose 90-day Oral Toxicity Study in Rodents” [24]. The experimental protocols were registered under the number 2016-003-2.

### 2.4 Preparation of test solutions

BBL powder was suspended at concentrations of 500, 1000, and 2500 mg/10 mL of 0.5% carboxymethyl cellulose-containing deionized water. The solutions were mixed and sonicated for 1 min prior to administering a dose of 10 mL/kg body weight.

### 2.5 Animals and treatments

Forty 4-week-old Sprague-Dawley rats of each sex were obtained from Japan SLC (Shizuoka, Japan). The animals were housed singly in stainless steel cages (260 mm × 380 mm × 200 mm) at 23 ± 2 °C, with 50 ± 10% humidity and a 12-h light/dark cycle (light: 9:00 AM to 9:00 PM) and free access to laboratory chow (MF; Oriental Yeast Company, Ltd., Tokyo, Japan) and deionized water. After 1 week of acclimatization, the animals were randomly divided into four groups. Three groups were orally administered the BBL solution once daily during the middle of the light period. Each group received either 2500 mg/kg (high dose), 1000 mg/kg (medium dose), or 500 mg/kg (low dose). The fourth group received 10 mL/kg of the vehicle.

### 2.6 Clinical and physiological observations

All animals were observed twice daily for mortality, general condition, and clinical signs. Any abnormal findings were recorded with respect to symptom, extent, severity, and date of detection. Body weights were measured daily, immediately prior to administration, and food consumption was measured three times per week. Water consumption was measured on week 12. The effect on the locomotor activity was also evaluated according to our previous reports [25]. Briefly, the rats were placed in open-field space (60 cm × 90 cm), and observed the locomotion and rearing frequency with in 5-min.

### 2.7 Urinalysis

Each rat was housed individually in a metabolic cage during week 10, and urine was collected over a period of 24 h. Urine volume was calculated using weight and density, analyzed by a urine specific gravity refractometer (MASTER-SUR/JM, Atago Co., Ltd., Tokyo, Japan). The color and turbidity were evaluated visually. Urinary glucose, total protein, and creatinine levels were analyzed using a Dri-Chem 4000v chemistry analyzer (Fujifilm Co., Tokyo, Japan). Urinary pH was measured with a pH meter (LAQUAtwin pH-11B, HORIBA Ltd., Kyoto, Japan). Urinary hemoglobin was analyzed with a hemoglobin B test kit (Wako Pure Chemical Industries Ltd.)

### 2.8 Hematology and blood chemistry

On week 13, the rats were fasted for 12 h and blood samples were taken from the abdominal vein as far as our possible under anesthesia with isoflurane. A 2-mL aliquot was added to K2-EDTA Venoject tubes (VP-DK052K05, Terumo Medical Corp., Tokyo, Japan). The samples were then stored at 4 °C and transported to LSI Medience Co. (Tokyo, Japan) for analysis of hematological parameters. Next, another aliquot of blood (6 mL) was added to Venoject tubes containing a procoagulant (VP-AL076 K, Terumo Medical Corp.) After staining for 30 min at 37 °C, the serum fraction was obtained by centrifugation (1200×*g*, 10 min, 15 °C) and stored at −80 °C until analysis. Serum biochemical parameters were analyzed using a Dri-Chem 4000v chemistry analyzer.

### 2.9 Necropsy and organ weights

After blood collection, the following organs and tissues were evaluated macroscopically and any abnormalities were recorded: Adrenal gland, duodenum, epididymis, eyes, heart, ileum, jejunum, kidneys, liver, lungs, ovaries, prostate, pancreas, skeletal muscle, skin, spleen, stomach, urinary bladder, uterus, testes, thymus, and thyroid. The following organs and tissues were weighed: Adrenal gland, heart, kidney, liver, lung, spleen, thymus, thyroid, and mesenteric visceral fat. All collected tissues were fixed in 10% neutral buffered formalin.

### 2.10 Histopathology

Tissue samples identified as possibly abnormal were also subjected to histological processing. The fixed thymus samples were transferred to New Histo Science Laboratory Co., Ltd. (Tokyo, Japan). The samples were stained with hematoxylin and eosin and then evaluated by pathologists.

### 2.11 Determination of serum cytokine/chemokine and corticosterone levels

We used a multiplex biometric enzyme-linked immunosorbent assay containing a dye microsphere conjugate with a monoclonal antibody specific for each target protein according to the manufacturer’s instructions (Rat Cytokine/Chemokine 10-Panel, RECYTMAG-65K-10, Millipore, Billerica, MA, USA), for the simultaneous detection and quantitation of interleukin (IL) 1α, IL-1β, IL-2, IL-6, interferon (IFN) γ, tumor necrosis factor (TNF) α, macrophage inflammatory protein (MIP) 1α, MIP-2, granulocyte colony-stimulating factor (G-CSF), and granulocyte-macrophage colony-stimulating factor (GM-CSF). Cytokine levels were determined using a multi-analyte profile with MAGPIX (Millipore). Serum corticosterone levels were determined with an enzyme immunoassay kit (Enzo Biochem Inc., Farmingdale, NY, USA).

### 2.12 Statistical analysis

Statistical analyses were conducted using StatView for Windows (version 5.0, SAS Institute, Cary, NC, USA). One-way analysis of variance was used for groups stratified by sex. If significant, Dunnett’s test for multiple comparisons was applied to compare the control and treatment groups. Results were considered significant if the probability of error was <5%.

## 3 Results

### 3.1 Mortality and clinical signs

BBL powder was orally administered daily for 90 days. The treatment appeared to be well-tolerated. No rat died during the exposure period, and no clinical signs such as diarrhea, hair loss or aberrant locomotion were observed.

### 3.2 Body weight and food consumption

Body weight gain did not differ between male and female rats in the treatment and control groups throughout the study period (Table 1). Mild, sporadic alterations in body weight gain were observed in male rats in the high-dose group on weeks 9 and 10. There was no statistically significant difference in food consumption between female rats in the treatment groups and control group, although significant changes were observed in male rats in the treatment groups on week 9 (Table 2).

**Table 1:** Mean body weight of rats administered blueberry leaf powder for 13 weeks.

Weeks	Males (n = 10)				Females (n = 10)			
	Control	500	1000	2500	Control	500	1000	2500
0	201.3 ± 9.1	201.5 ± 9.0	201.1 ± 8.5	200.5 ± 9.3	144.3 ± 14.0	144.8 ± 8.0	144.5 ± 6.1	145.2 ± 9.2
1	229.1 ± 14.3	228.5 ± 12.3	229.5 ± 12.3	226.6 ± 13.0	156.9 ± 12.7	158.4 ± 7.2	158.8 ± 6.8	160.1 ± 13.3
2	282.3 ± 19.9	277.8 ± 19.4	282.5 ± 19.1	276.9 ± 18.0	179.4 ± 15.7	180.1 ± 9.2	181.0 ± 11.3	184.5 ± 16.8
3	330.9 ± 25.2	324.4 ± 19.4	331.7 ± 23.9	324.3 ± 21.6	200.6 ± 18.9	199.6 ± 10.6	200.4 ± 13.9	206.1 ± 19.5
4	370.3 ± 31.5	362.9 ± 22.8	371.9 ± 26.9	362.6 ± 27.7	217.0 ± 20.5	216.1 ± 11.3	217.2 ± 16.7	222.6 ± 20.2
5	401.4 ± 34.3	396.0 ± 24.4	402.1 ± 29.5	388.2 ± 31.8	230.2 ± 20.4	226.5 ± 12.1	229.2 ± 19.4	235.2 ± 21.3
6	431.9 ± 39.0	424.2 ± 30.7	429.2 ± 37.4	411.9 ± 38.3	240.8 ± 22.5	235.8 ± 13.9	237.3 ± 16.7	244.2 ± 22.6
7	452.1 ± 41.7	442.6 ± 36.1	445.1 ± 35.6	423.8 ± 40.7	249.6 ± 23.2	246.3 ± 16.5	247.7 ± 16.4	252.3 ± 21.6
8	470.1 ± 44.6	458.5 ± 40.0	458.4 ± 40.5	437.3 ± 41.9	254.6 ± 23.1	254.5 ± 19.0	252.7 ± 16.2	259.5 ± 24.5
9	482.5 ± 48.7	452.8 ± 41.7	453.9 ± 41.2	440.3 ± 46.7	259.9 ± 23.9	261.7 ± 21.0	256.9 ± 16.4	264.7 ± 24.8
10	485.1 ± 49.4	440.9 ± 37.2	446.8 ± 36.4	441.1 ± 50.3	265.6 ± 23.6	266.7 ± 21.3	259.5 ± 16.4	265.7 ± 22.2
11	490.8 ± 54.1	458.2 ± 40.0	468.1 ± 38.3	456.9 ± 49.7	270.1 ± 24.7	269.8 ± 22.4	264.4 ± 18.3	267.0 ± 25.4
12	491.9 ± 56.8	464.5 ± 43.7	476.2 ± 43.2	462.8 ± 51.2	274.9 ± 25.7	273.3 ± 23.6	268.0 ± 17.4	272.5 ± 25.2
13	498.7 ± 55.8	472.7 ± 41.7	479.3 ± 48.7	472.2 ± 53.9	277.4 ± 27.2	274.9 ± 24.8	269.7 ± 19.9	275.1 ± 24.4

All values represent the mean (in grams) ± S.D. No significant differences were found between control and treated rats (*P* <  0.05, Dunnett’s test).

**Table 2:** Mean food consumption by rats administered blueberry leaf powder for 13 weeks.

Weeks	Dose groups (mg/kg body weight/day)							
	Males (n = 10)				Females (n = 10)			
	Control	500	1000	2500	Control	500	1000	2500
0	19.4 ± 2.7	19.7 ± 2.4	19.6 ± 2.0	19.8 ± 2.0	14.5 ± 1.3	14.5 ± 1.2	15.3 ± 1.0	15.4 ± 1.4
1	20.6 ± 2.0	20.4 ± 2.1	20.6 ± 2.1	21.0 ± 1.7	14.7 ± 1.6	15.1 ± 1.0	15.3 ± 1.3	15.8 ± 1.5
2	23.0 ± 2.2	22.7 ± 2.6	23.5 ± 2.3	23.0 ± 2.4	15.8 ± 1.7	15.3 ± 1.4	15.9 ± 1.6	16.4 ± 1.9
3	24.7 ± 2.0	23.8 ± 2.9	24.8 ± 2.1	24.5 ± 2.5	15.9 ± 2.2	15.7 ± 1.3	16.2 ± 1.8	16.7 ± 1.6
4	24.5 ± 2.1	24.3 ± 1.8	24.4 ± 3.1	23.0 ± 2.4	16.1 ± 1.9	17.5 ± 4.8	16.3 ± 1.7	16.6 ± 1.6
5	23.7 ± 2.5	24.0 ± 1.8	24.0 ± 2.5	22.5 ± 2.3	16.0 ± 1.5	15.3 ± 1.4	15.7 ± 2.2	15.9 ± 2.2
6	24.2 ± 2.7	23.8 ± 3.5	24.0 ± 3.9	21.0 ± 2.8	15.4 ± 2.8	15.3 ± 2.5	15.7 ± 1.6	15.7 ± 1.7
7	22.7 ± 2.9	23.0 ± 2.7	22.2 ± 4.6	20.7 ± 1.5	15.7 ± 1.9	16.2 ± 2.4	16.2 ± 1.4	15.8 ± 1.5
8	22.5 ± 3.3	21.7 ± 3.2	21.2 ± 3.8	20.6 ± 2.1	14.8 ± 1.7	16.1 ± 2.3	15.3 ± 1.7	16.0 ± 1.9
9	22.6 ± 3.2	17.4 ± 3.9*	17.2 ± 3.2*	17.2 ± 1.4*	15.2 ± 1.8	16.4 ± 1.7	15.5 ± 1.6	16.1 ± 1.3
10	21.6 ± 2.9	19.1 ± 3.7	20.2 ± 2.5	20.9 ± 3.5	15.1 ± 1.3	14.8 ± 2.3	14.8 ± 1.8	13.5 ± 2.4
11	19.6 ± 3.3	20.2 ± 2.9	21.1 ± 3.1	20.9 ± 2.8	15.1 ± 1.3	14.8 ± 1.8	14.3 ± 1.3	14.5 ± 2.8
12	19.6 ± 3.2	19.4 ± 1.7	20.1 ± 3.1	20.0 ± 2.7	14.8 ± 1.7	14.8 ± 2.4	14.5 ± 1.4	14.4 ± 2.4
13	20.2 ± 2.7	18.9 ± 1.1	20.0 ± 3.4	20.0 ± 2.5	15.2 ± 1.5	14.6 ± 2.6	14.6 ± 1.2	15.2 ± 2.0

All values represent the mean (in grams) ± S.D. *Significantly different from the controls (*P* <  0.05, Dunnett’s test).

### 3.3 Hematology and blood chemistry

White blood cell (WBC) counts and eosinophil percentage significantly increased in the low-dose male group (Table 3). Neutrophil percentage increased and lymphocyte percentage significantly decreased in the medium-dose male group. Other blood chemistry parameters in male groups and all parameters in female groups were not altered at the end of the 90-day exposure period.

**Table 3:** Hematological parameters in rats administered blueberry leaf powder for 13 weeks.

Weeks	Dose groups (mg/kg body weight/day)								
	Males					Females			
	Control (n = 9)^1^	500 (n = 10)	1000 (n = 10)	2500 (n = 10)		Control (n = 10)	500 (n = 9)^1^	1000 (n = 10)	2500 (n = 10)
WBC (10^3^/μL)	7.19 ± 0.68	8.83 ± 1.43*	7.62 ± 1.87	7.31 ± 0.96		5.71 ± 1.55	6.51 ± 0.73	6.64 ± 2.17	6.30 ± 1.21
RBC (10^6^/μL)	8.90 ± 0.30	9.14 ± 0.33	8.88 ± 0.28	8.99 ± 0.30		8.48 ± 0.38	8.49 ± 0.29	8.54 ± 0.30	8.26 ± 0.42
HGB (g/dL)	14.4 ± 0.6	14.9 ± 0.5	14.4 ± 0.4	14.5 ± 1.0		14.9 ± 0.3	15.2 ± 0.4	15.1 ± 0.5	14.6 ± 1.0
HCT (%)	42.3 ± 1.4	42.1 ± 1.5	41.5 ± 1.4	42.8 ± 1.6		46.2 ± 1.5	46.6 ± 1.7	46.7 ± 1.9	44.6 ± 2.9
MCV (fL)	47.4 ± 1.4	46.1 ± 1.4	46.8 ± 1.0	47.8 ± 0.9		54.7 ± 2.6	55.2 ± 1.9	54.7 ± 1.9	53.9 ± 2.0
MCH (pg)	16.2 ± 0.5	16.3 ± 0.9	16.2 ± 0.2	16.1 ± 1.1		17.6 ± 0.4	17.9 ± 0.4	17.7 ± 0.3	17.6 ± 0.5
MCHC (g/dL)	0.34 ± 0.01	0.35 ± 0.02	0.35 ± 0.01	0.34 ± 0.03		0.32 ± 0.01	0.33 ± 0.01	0.32 ± 0.01	0.33 ± 0.01
PLT (10^4^/μL)	8.71 ± 1.85	8.97 ± 1.77	8.82 ± 1.68	8.18 ± 1.55		7.82 ± 1.78	8.02 ± 1.14	7.67 ± 1.11	7.97 ± 1.93
Leukocyte fractionation (%)									
Neutrophil	14.5 ± 2.5	18.2 ± 4.7	21.8 ± 5.9*	19.1 ± 8.3	17.6 ± 3.9		19.1 ± 7.7	25.4 ± 9.6	25.6 ± 7.3
Lymphocyte	80.2 ± 3.2	74.8 ± 6.1	71.8 ± 6.5*	74.7 ± 10.1	76.6 ± 5.7		75.0 ± 9.4	68.1 ± 10.4	68.8 ± 8.0
Monocyte	2.93 ± 0.57	2.90 ± 0.78	2.99 ± 0.39	3.75 ± 1.32	2.87 ± 0.81		2.34 ± 0.41	2.53 ± 0.42	2.78 ± 0.77
Eosinophil	2.34 ± 1.10	4.01 ± 1.48*	3.45 ± 1.23	2.52 ± 1.53	2.78 ± 1.35		3.52 ± 1.82	3.84 ± 1.99	2.80 ± 1.33
Basophil	0.04 ± 0.05	0.03 ± 0.05	0.03 ± 0.07	0.01 ± 0.03	0.16 ± 0.33		0.06 ± 0.08	0.09 ± 0.19	0.06 ± 0.08

All values represent the mean ± S.D. HCT, hematocrit; HGB, hemoglobin; MCH, mean corpuscular hemoglobin; MCHC, mean corpuscular hemoglobin concentration; MCV, mean corpuscular volume; PLT, platelet; RBC, red blood cell; WBC, white blood cell.1One data was missing, because one blood sample was coagulated before analysis. No significant differences were found between control and treated rats (P <  0.05, Dunnett’s test).

Serum biochemistry assessment revealed significantly changes in aspartate aminotransferase activity and potassium amount in the male high-dose group (Table 4). These changes were not observed in the female groups. Other parameters were not altered at the end of the 90-day exposure period.

**Table 4:** Serum biochemistry parameters in rats administered blueberry leaf powder for 13 weeks.

Weeks	Dose groups (mg/kg body weight/day)							
	Males (n = 10)				Females (n = 10)			
	Control	500	1000	2500	Control	500	1000	2500
ALT (U/L)	25.7 ± 4.3	25.2 ± 2.8	24.8 ± 3.2	25.7 ± 3.8	45.2 ± 23.6	37.1 ± 12.4	37.9 ± 10.9	37.7 ± 11.6
AST (U/L)	60.9 ± 14.0	51.7 ± 7.0	53.9 ± 9.8	49.5 ± 5.0*	99.0 ± 54.6	78.8 ± 18.5	85.5 ± 27.9	76.0 ± 11.1
Alkaline phosphatase (U/L)	376 ± 82	360 ± 70	427 ± 191	439 ± 123	409 ± 148	386 ± 106	347 ± 114	433 ± 162
Amylase (kU/L)	2.04 ± 1.53	1.89 ± 1.21	1.96 ± 2.02	1.87 ± 2.18	1.36 ± 3.26	1.34 ± 1.84	1.29 ± 2.23	1.31 ± 2.28
Leucine aminopeptidase (U/L)	57.0 ± 4.6	58.8 ± 4.6	57.3 ± 4.0	57.0 ± 3.7	60.9 ± 5.9	59.8 ± 5.9	57.1 ± 7.0	57.5 ± 4.1
LDH (U/L)	338 ± 105	298 ± 138	307 ± 132	307 ± 111	659 ± 120	650 ± 198	669 ± 194	545 ± 209
Creatine phosphokinase (U/L)	111 ± 27	111 ± 45	94 ± 25	99 ± 30	165 ± 27	173 ± 71	177 ± 50	135 ± 47
Total bilirubin (mg/dL)	0.18 ± 0.05	0.16 ± 0.03	0.20 ± 0.05	0.17 ± 0.04	0.27 ± 0.08	0.27 ± 0.07	0.23 ± 0.08	0.25 ± 0.06
Glucose (mg/dL)	167 ± 11	160 ± 24	164 ± 22	151 ± 15	103 ± 20	106 ± 27	97 ± 28	112 ± 17
Blood urea nitrogen (mg/dL)	13.7 ± 1.3	13.0 ± 1.0	12.2 ± 2.0	12.5 ± 2.7	14.1 ± 4.1	14.1 ± 2.7	13.7 ± 4.3	13.7 ± 3.2
Creatinine (mg/dL)	0.29 ± 0.03	0.27 ± 0.06	0.27 ± 0.06	0.28 ± 0.07	0.23 ± 0.04	0.24 ± 0.06	0.22 ± 0.03	0.23 ± 0.04
Total cholesterol (mg/dL)	47.7 ± 6.1	53.8 ± 11.2	46.5 ± 9.8	43.7 ± 11.3	80.1 ± 22.1	73.6 ± 22.0	66.8 ± 22.5	73.9 ± 21.4
Triglycerides (mg/dL)	70.3 ± 28.5	78.7 ± 40.9	61.7 ± 17.3	58.6 ± 20.8	42.0 ± 19.0	38.6 ± 9.4	40.2 ± 16.4	38.5 ± 20.8
Albumin (g/dL)	2.87 ± 0.34	2.78 ± 0.43	2.73 ± 0.55	2.52 ± 0.50	3.42 ± 0.69	3.23 ± 0.35	3.08 ± 0.69	3.30 ± 0.44
Total protein (g/dL)	5.85 ± 0.26	5.91 ± 0.25	5.94 ± 0.23	5.70 ± 0.18	5.57 ± 0.87	5.37 ± 0.58	5.17 ± 1.00	5.54 ± 0.62
NH_3_ (μg/dL)	148 ± 17	138 ± 11	134 ± 31	127 ± 19	119 ± 29	116 ± 26	109 ± 22	121 ± 16
Phosphorus (mg/dL)	5.55 ± 0.64	5.54 ± 0.48	5.51 ± 0.53	5.57 ± 0.94	4.64 ± 0.72	4.52 ± 0.89	4.07 ± 0.70	4.78 ± 1.33
Calsium (mg/dL)	8.41 ± 0.79	8.42 ± 0.73	8.25 ± 0.90	7.74 ± 1.05	8.45 ± 1.36	8.35 ± 1.05	7.97 ± 1.61	8.28 ± 0.68
Magnesium (mg/dL)	1.99 ± 0.18	1.92 ± 0.20	1.87 ± 0.21	1.83 ± 0.28	1.95 ± 0.28	2.01 ± 0.18	1.90 ± 0.37	2.02 ± 0.24
Sodium (mEq/L)	141 ± 2	140 ± 1	140 ± 2	140 ± 2	137 ± 2	139 ± 2	139 ± 3	137 ± 5
Potassium (mEq/L)	4.52 ± 0.18	4.40 ± 0.19	4.43 ± 0.17	4.34 ± 0.12*	3.92 ± 0.33	3.89 ± 0.24	4.02 ± 0.28	3.97 ± 0.29
Chloride (mEq/L)	100 ± 2	100 ± 1	100 ± 3	100 ± 1	99 ± 3	100 ± 3	98 ± 5	99 ± 4

All values represent the mean ± S.D. ALT, alanine aminostransferase; AST, aspartate aminotransferase; LDH, lactate dehydrogenase.

No significant differences were found between control and treated rats (P <  0.05, Dunnett’s test).

### 3.4 Urinalysis

No significant variations in pH, total protein, or glucose levels were found by urinalysis in any treatment group (Table 5).

**Table 5:** Urinalysis findings in rats administered blueberry leaf powder for 13 weeks.

Weeks	Dose groups (mg/kg body weight/day)							
	Males (n = 10)				Females (n = 10)			
	Control	500	1000	2500	Control	500	1000	2500
pH	6.86 ± 0.05	6.84 ± 0.07	6.85 ± 0.10	6.84 ± 0.05	6.85 ± 0.05	6.95 ± 0.12	6.88 ± 0.10	6.79 ± 0.14
Total protein (g/g creatinine)								
	3.02 ± 1.51	3.39 ± 2.12	3.31 ± 1.70	3.67 ± 2.55	4.16 ± 2.11	4.14 ± 1.68	4.42 ± 1.00	4.03 ± 1.33
Glucose (g/g creatinine)								
	0.18 ± 0.13	0.14 ± 0.07	0.24 ± 0.18	0.15 ± 0.06	0.08 ± 0.06	0.06 ± 0.05	0.09 ± 0.11	0.10 ± 0.05

All values represent the mean ± S.D. No significant differences were found between control and treated rats (P <  0.05, Dunnett’s test).

### 3.5 Necropsy

No visible alterations were associated with BBL treatment, with the exception of protruding teeth in one female rat in the control group. Sporadic findings included black spots on the spleen of some rats in the control and treatment groups; these findings were not associated with the dose.

### 3.6 Organ weights

There were no significant differences in absolute and relative organ weights between sexes or treatment groups, although thymus weights in both male and female rats tended to decrease as the BBL dose increased (Table 6).

**Table 6:** Absolute and relative organ weights in rats administered blueberry leaf powder for 13 weeks.

	Dose groups (mg/kg body weight/day)							
	Males (n = 10)				Females (n = 10)			
	Control	500	1000	2500	Control	500	1000	2500
Absolute organ weights (g)								
Body weights	481 ± 50	453 ± 41	462 ± 48	442 ± 38	266 ± 28	265 ± 24	259 ± 20	265 ± 22
Liver	12.1 ± 1.6	11.3 ± 1.1	11.9 ± 1.9	11.0 ± 1.2	6.8 ± 0.4	6.4 ± 0.7	6.6 ± 0.6	6.9 ± 0.5
Kidney	2.69 ± 0.28	2.65 ± 0.42	2.66 ± 0.24	2.58 ± 0.22	1.62 ± 0.15	1.55 ± 0.14	1.55 ± 0.12	1.62 ± 0.12
Adrenal	0.057 ± 0.015	0.055 ± 0.011	0.062 ± 0.013	0.056 ± 0.010	0.068 ± 0.010	0.069 ± 0.009	0.068 ± 0.013	0.072 ± 0.010
Spleen	0.81 ± 0.08	0.74 ± 0.11	0.79 ± 0.10	0.78 ± 0.08	0.51 ± 0.07	0.51 ± 0.06	0.54 ± 0.06	0.54 ± 0.07
Heart	1.28 ± 0.12	1.22 ± 0.12	1.25 ± 0.11	1.21 ± 0.08	0.78 ± 0.07	0.72 ± 0.06	0.75 ± 0.04	0.75 ± 0.04
Lung	1.46 ± 0.13	1.51 ± 0.17	1.52 ± 0.11	1.40 ± 0.09	1.03 ± 0.05	1.04 ± 0.06	1.06 ± 0.07	1.06 ± 0.08
Visceral fat^1^	7.9 ± 2.4	7.5 ± 2.5	7.1 ± 2.4	6.1 ± 2.1	5.19 ± 2.40	6.10 ± 5.14	6.10 ± 4.84	4.18 ± 1.98
Thymus	0.440 ± 0.076	0.366 ± 0.090	0.389 ± 0.093	0.343 ± 0.05	0.324 ± 0.030	0.300 ± 0.048	0.285 ± 0.047	0.270 ± 0.051
Thyroid	0.015 ± 0.002	0.014 ± 0.004	0.014 ± 0.002	0.014 ± 0.003	0.010 ± 0.003	0.011 ± 0.002	0.012 ± 0.003	0.012 ± 0.003
Relative organ weight (g/100 g body weight)								
Liver	2.51 ± 0.18	2.48 ± 0.11	2.56 ± 0.21	2.49 ± 0.13	2.58 ± 0.15	2.42 ± 0.16	2.54 ± 0.16	2.60 ± 0.17
Kidney	0.561 ± 0.048	0.585 ± 0.076	0.578 ± 0.025	0.583 ± 0.032	0.612 ± 0.049	0.587 ± 0.042	0.599 ± 0.029	0.613 ± 0.058
Adrenal	0.012 ± 0.003	0.012 ± 0.003	0.014 ± 0.003	0.013 ± 0.002	0.025 ± 0.003	0.026 ± 0.005	0.026 ± 0.005	0.027 ± 0.004
Spleen	0.169 ± 0.015	0.162 ± 0.013	0.172 ± 0.021	0.177 ± 0.012	0.191 ± 0.021	0.194 ± 0.030	0.210 ± 0.019	0.203 ± 0.027
Heart	0.267 ± 0.015	0.269 ± 0.015	0.272 ± 0.017	0.275 ± 0.020	0.295 ± 0.024	0.274 ± 0.016	0.290 ± 0.013	0.285 ± 0.023
Lung	0.305 ± 0.019	0.335 ± 0.031	0.331 ± 0.029	0.318 ± 0.025	0.392 ± 0.035	0.394 ± 0.037	0.411 ± 0.049	0.404 ± 0.038
Visceral fat^1^	1.65 ± 0.49	1.64 ± 0.48	1.52 ± 0.46	1.37 ± 0.39	1.90 ± 0.70	2.24 ± 1.75	2.30 ± 1.72	1.55 ± 0.61
Thymus	0.092 ± 0.017	0.080 ± 0.016	0.084 ± 0.17	0.077 ± 0.008*	0.122 ± 0.011	0.114 ± 0.019	0.110 ± 0.015	0.103 ± 0.022*
Thyroid	0.0031 ± 0.0005	0.0032 ± 0.0010	0.0030 ± 0.0004	0.0032 ± 0.0007	0.0039 ± 0.0011	0.0040 ± 0.0007	0.0045 ± 0.0015	0.0047 ± 0.0012

All values represent the mean ± S.D. ^1^Mesenteric fat. No significant differences were found between control and treated rats (*P* <  0.05, Dunnett’s test).

### 3.7 Histopathology

**Fig. 2:**
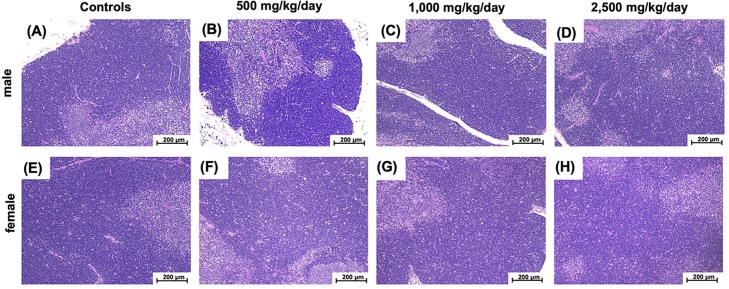
Light microscopy of rat thymus tissues stained with hematoxylin and eosin (×10). Blueberry leaf powder was orally administered to male (A–D) and female (E–H) rats daily for 90 days. (A, E) Vehicle solvent (controls); (B, F) 500 mg/kg/day; (C, G) 1000 mg/kg/day; (D, H) 2500 mg/kg/day.

Histopathological examination of the thymus did not reveal any changes in any group (Fig. 2).

### 3.8 Serum cytokine/chemokine and corticosterone levels

Table 7 shows the serum levels of various cytokines and corticosterone in male and female rats following a 90-day BBL treatment. The values measured for IL-1α, IL-1β, IFN-γ, MIP-2, G-CSF, and GM-CSF were below the detection limits in some animals and did not exhibit a dose-response relationship. Therefore, the data are given as the median (minimum–maximum). Notable alterations were not observed in any treatment group or either sex.

**Table 7:** Cytokine and corticosterone serum levels in rats administered blueberry leaf powder for 13 weeks.

Weeks	Dose groups (mg/kg body weight/day)							
	Males (n = 10)				Females (n = 10)			
	Control	500	1000	2500	Control	500	1000	2500
G-CSF	68.9 (<4.9ー108.4)	62.4 (27.9ー97.6)	62.4 (22.3ー90.9)	73.3 (25.1ー108.4)	37.9 (10.1ー77.4)	67.7 (43.0ー100.1)	49.2 (14.1ー103.3)	68.6 (21.9ー121.0)
GM-CSF	12.2 (<12.2ー37.3)	12.2 (<12.2)	12.2 (<12.2)	12.2 (<12.2)	12.2 (<12.2)	12.2 (<12.2ー17.6)	12.2 (<12.2)	12.2 (<12.2)
MIP-1α	27.7 (15.7ー45.4)	21.6 (13.5ー27.8)	22.2 (17.6ー36.0)	29.9 (15.7ー35.7)	28.7 (19.3ー33.5)	26.4 (22.5ー31.5)	27.4 (22.8ー33.5)	26.3 (23.5ー28.8)
MIP-2	24.6 (<24.4ー112.4)	24.4 (<24.4ー46.3)	24.4 (<24.4ー36.7)	24.4 (<24.4ー36.6)	24.4 (<24.4ー36.6)	24.4 (<24.4ー66.3)	24.4 (<24.4ー36.0)	24.4 (<24.4ー83.0)
IL-1α	82.5 (<12.2ー141.8)	51.1 (<12.2ー111.0)	72.3 (12.3ー111.1)	72.9 (<12.2ー103.3)	20.4 (14.8ー33.9)	22.0 (8.2ー32.3)	25.2 (11.5ー37.0)	30.7 (16.4ー43.2)
IL-1β	40.9 (3.4ー66.1)	26.7 (8.4ー53.1)	29.4 (<2.4ー43.4)	31.0 (6.3ー51.5)	7.5 (2.1ー36.4)	10.3 (4.9ー16.4)	12.7 (3.5ー35.0)	11.9 (8.1ー20.5)
IL-2	140.5 (10.9ー231.4)	104.8 (16.3ー180.9)	129.0 (61.1ー182.3)	132.8 (19.0ー256.5)	69.8 (52.0ー127.3)	90.6 (52.0ー154.2)	94.0 (24.1ー196.2)	110.4 (51.3ー175.7)
IL-6	1848.3 (92.4ー2993.3)	1356.5 (363.2ー2218.8)	1481.7 (360.7ー2402.3)	1495.8 (232.4ー1848.0)	251.0 (<73.2ー835.2)	488.5 (<73.2ー979.2)	440.1 (<73.2ー979.2)	874.5 (176.7ー133.9)
IFNγ	14.6 (<14.6ー123.2)	14.6 (<14.6ー210.2)	14.6 (<14.6ー289.6)	25.2 (<14.6ー344.7)	14.6 (<14.6)	14.6 (<14.6)	14.6 (<14.6)	14.6 (<14.6)
TNFα	14.2 (2.1ー22.6)	10.7 (2.5ー18.9)	11.4 (4.9ー15.1)	10.9 (3.2ー18.9)	2.6 (2.3ー3.2)	2.8 (2.3ー4.0)	2.8 (2.3ー3.9)	3.2 (2.2ー4.1)
Corticosterone	87.8 (81.9ー93.0)	89.4 (84.6ー93.1)	91.2 (79.0ー93.1)	88.7 (81.9ー92.8)	111.7 (107.3ー113.9)	113.1 (110.4ー115.2)	112.6 (109.0ー116.1)	111.1 (108.2ー112.9)

All values represent the median (min–max).

## 4 Discussion

Berry fruits such as blueberries are considered functional foods because of their high contents of natural antioxidants [26]. The leaves of these fruits have garnered attention as potential functional foods as well, although safety information on berry leaf consumption is limited [19,20]. We conducted this study to evaluate the effects of consuming rabbiteye BBL powder. There were no deaths or changes in behavior or external appearance among the rats dosed daily with BBL for 90 days. No significant alterations in hematological and serum chemical parameters, urinalysis, food consumption, body weight gain, or absolute and relative organ weights were noted in any of the dose groups, with a few exceptions. Otherwise, some other parameters listed in OECD guideline [24] including weights of organs such as brain, spinal cord and lungs, and some behaviors such as functional observational battery were not evaluated in this study. Therefore, effects of consuming rabbiteye BBL powder on these parameters were remained as our future study for evaluation.

A reduction in food consumption and body weight gain were observed in the male high-dose group on weeks 9 and 10. In a previous study, male rats administered γ-aminobutyric acid exhibited a marked decrease in body weight gain and food consumption during the middle of the administration period [21]; the researchers concluded that the finding was not toxicologically relevant because body weight gain at the end of the treatment period did not vary from the gain observed in the controls.

Higher white blood cell counts and eosinophil percentage were noted in the male low-dose group, and higher neutrophil percentage and lower lymphocyte percentage were noted in the male medium-dose group. An increase in white blood cell counts is recognized as immune stimulation, and typically occurs concomitantly with an increase in IL-1β and TNF-α production and a decrease in IL-6 production [27]. In our study, however, we did not observe alterations in cytokine/chemokine production after 90 days of daily treatment. Serum aspartate aminotransferase activity in the high-dose group decreased in both male and female rats. Both aspartate aminotransferase and alanine aminotransferase are secreted from hepatic cells into the bloodstream following liver injury [28]; we did not observe changes in serum alanine aminotransferase activity in any of the dose groups. The findings did not indicate a dose-response relationship and were sporadic but not toxic.

A non-significant dose-response relationship was observed in thymus weights in both sexes. Chronic physiological and/or social stress can induce thymic atrophy, adrenal hypertrophy, and over-secretion of corticosterone [29]. We did not observe changes in adrenal gland weight or serum corticosterone in either sex or at any dose. Additionally, no remarkable histopathological findings were observed in the thymus.

In conclusion, a 90-day treatment with rabbiteye BBL powders appeared to be well-tolerated in Sprague-Dawley rats. No significant changes in clinical signs, hematology, blood chemistry, urinalysis, or histopathology were observed. Many 90-day repeated dose studies using rodents suggested a no-observed-adverse-effect level (NOAEL) according to the results obtained [30]. Our results indicated that a daily dose up to 2500 mg of BBL powder per kg body weight may therefore be a NOAEL. Acceptable daily intake represents a level of exposure “without appreciable health risk’’ for humans when consumed daily or weekly over a lifetime, and is derived by applying an uncertainty factor of 100 to the NOAEL [31]. Therefore, the acceptable daily intake of BBL powder for humans is 25 mg in dry weight (ca. 100 mg in wet weight) per body weight. Recent studies have reported that leaves such as pumpkin, nutmeg and curry might exert several beneficial effects including anti-gastro toxic and anti-cholinesterase [[32], [33], [34]]. Therefore, we consider BBL powder also be another candidate for beneficial food material. To be a functional food, BBL powder must be shown to contain beneficial compounds. The next logical step is to characterize the detail constituents of whole BBL powder chemically in addition of phenolic acids, flavonoids and procyanidins, and then to design studies to evaluate if the administration improves certain health conditions or prevents the onset of adverse health conditions including diabetes, kidney disease and metabolic disorders.

## Transparency document

Transparency document

## Conflict of interest

The authors hereby state that there is no conflict of interest or any contractual relations or proprietary considerations that would affect the publication of the information in this article.
